# Uncertainty-Aware Probabilistic Fusion Post-Processing for Continuous Wrist Motion Estimation in Myoelectric Control

**DOI:** 10.3390/s26092614

**Published:** 2026-04-23

**Authors:** Sheng Feng, Guangyong Xu, Yinglin Li

**Affiliations:** 1 Sichuan Jiuzhou Electric Group Co., Ltd., Mianyang 621100, China; shengf1984@163.com (S.F.); guangyongx@126.com (G.X.); 2 School of Marine Science and Technology, Northwestern Polytechnical University, Xi’an 710072, China

**Keywords:** surface electromyography (sEMG), continuous joint motion estimation, post-processing, myoelectric control, local Gaussian process regression, probabilistic fusion

## Abstract

Continuous wrist angle estimation based on surface electromyography (sEMG) is often affected by signal variability and prediction instability. Although regression models provide instantaneous outputs, their predictions may exhibit temporal fluctuations and limited robustness due to the non-stationary nature of sEMG signals. To address this issue, we propose an uncertainty-aware probabilistic fusion post-processing framework for continuous wrist motion estimation. The proposed approach decouples regression and uncertainty modeling, enabling plug-in compatibility with feature-based regression models. A local Gaussian process regression (LGPR) model is employed to estimate predictive uncertainty from a sliding feature window. The instantaneous regression output is then fused with the LGPR prediction through a Bayesian-inspired Gaussian formulation, resulting in a closed-form adaptive gain that dynamically adjusts smoothing strength according to predictive variance. Experimental results from both open-loop wrist joint motion estimation and closed-loop myoelectric control tasks demonstrate that our method outperforms existing methods in key performance indicators, including task completion time, trajectory smoothness, and trajectory tracking error.

## 1. Introduction

With the ongoing advancements in wearable devices and human–robot interaction technologies, surface electromyography (sEMG)-based joint motion estimation has found broad applications in fields such as rehabilitation medicine [1,2,3] and robotic control [4,5,6]. sEMG signals, which reflect the electrical activity of muscles, enable the prediction of human motion, facilitating continuous robot control [7,8]. As a result, improving the accuracy of joint motion estimation through sEMG decoding has become a critical research focus. Traditional estimation methods typically rely on model-based approaches [9,10] or direct sensor measurements [6,11,12]. However, these methods struggle in dynamic environments due to non-stationarity and noise. In contrast, with the rise of artificial intelligence, machine learning-based joint motion estimation has emerged as an effective complement [13].

Recent advancements in continuous joint motion estimation based on sEMG, particularly using regression models [1,14,15,16] or deep learning models [17,18,19], have enabled significant progress in mapping regression outcomes to robotic arms or prosthetics through simultaneous proportional control. For instance, random forest (RF) regression, a nonlinear method, has demonstrated the ability to handle high-dimensional features and provide accurate predictions [15,20]. However, these methods often rely on direct predictions from raw sEMG signals or extracted features, which can lead to substantial prediction errors and less smooth trajectories.

To improve stability and accuracy, several studies [21,22,23,24] have proposed post-processing methods that apply smoothing or filtering techniques after sEMG decoding to reduce noise and optimize control commands. Techniques such as weighted moving average filters [22,24,25] and wavelet transforms [26,27] effectively smooth estimates and reduce noise. While simple and effective, these methods typically use fixed weighting coefficients or sliding windows, limiting their adaptability to real-time prediction uncertainties [17]. As a result, their performance in dynamic conditions may be suboptimal, reducing their effectiveness in improving motion estimation accuracy.

**Figure 1:**
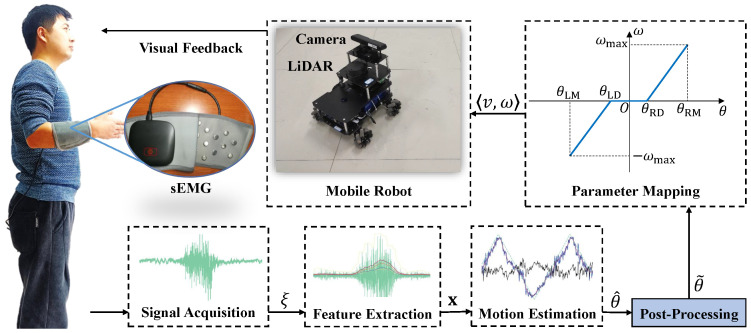
The myoelectric control system for continuous wrist joint motion estimation driven by sEMG signals.

This work focuses on continuous wrist joint motion estimation through sEMG signal decoding, using predicted wrist joint angles to generate motion commands for a mobile robotic platform, as illustrated in Figure 1. While accuracy is important in regression predictions, our primary emphasis is on the smoothness of predicted trajectories, noise reduction, and enhanced robustness, particularly during rapid gesture transitions [28]. The goal is to improve the continuity and stability of wrist joint angle predictions, thereby optimizing the accuracy and responsiveness of myoelectric control for robotic movement.

While direct kinematic sensing (e.g., IMU) suffices for intact-limb applications, EMG offers unique advantages in scenarios where joint motion is unavailable or invisible: prosthetic control for amputees, sub-threshold intention detection in neurorehabilitation, and fatigue-adaptive assistance. This study employs wrist angle as a validation platform to establish a generalizable decoding framework for these higher-impact applications.

To address these limitations, we propose an uncertainty-aware probabilistic fusion post-processing framework for continuous wrist angle estimation. Instead of treating smoothing as a heuristic filtering operation, the proposed method formulates temporal refinement as a Gaussian fusion problem that explicitly incorporates predictive uncertainty. A local Gaussian process regression (LGPR) model is employed to estimate predictive mean and variance from a sliding feature window, and the instantaneous regression output is fused with the LGPR prediction via a closed-form adaptive gain. This formulation enables confidence-aware smoothing while preserving plug-in compatibility with feature-based regression models.

The main contributions of this work are summarized as follows:

- We propose a practical uncertainty-aware post-processing mechanism that enables plug-and-play compatibility with existing regressors without retraining or architectural modification.
- We formulate post-processing as a Gaussian fusion problem with a closed-form adaptive gain, where predictive uncertainty dynamically regulates smoothing strength.
- We validate the method through open-loop wrist joint motion estimation and closed-loop myoelectric control experiments, demonstrating superior performance in task completion time, trajectory smoothness, and trajectory tracking error compared to traditional post-processing techniques.

The remainder of this paper is organized as follows. Section 2 describes the experimental apparatus and data acquisition procedures. Section 3 presents the proposed uncertainty-aware probabilistic fusion method in detail. Section 4 reports and analyzes the experimental results. Finally, Section 5 concludes the paper.

## 2. Apparatus and Experiments

### 2.1. Mobile Platform

A mobile robot is used as the platform for the robotic system. The robot is equipped with a camera, a laser range sensor, and an odometer, and it communicates with the human operator control platform via WiFi.

The kinematic model of the robot can be expressed as

(1) $$s_{t}=s_{t-1}+\Delta t\cdot u_{t}$$

where $s_{t}={\left[x_{t},y_{t},\theta_{t}\right]}^{⊤}\in S$ represents the robot’s state at time *t*, consisting of its 2D position ${\left[x,y\right]}^{⊤}$ and orientation angle θ. $u_{t}={\left[v_{t},\omega_{t}\right]}^{⊤}\in U$ represents the control input at time *t*, which includes the linear velocity *v* and angular velocity ω. Δt denotes the time step. In our implementation, the control frequency of the robot is 10 Hz.

### 2.2. Parameter Mapping

The wrist flexion–extension angle is mapped to the robot’s angular velocity control commands, directly driving its movement. An sEMG device worn on the left hand enables predefined control: wrist extension turns the robot left, wrist flexion turns it right, and a neutral wrist moves it straight.

The forearm axis serves as the zero reference for wrist flexion/extension, with flexion defined as positive and extension as negative. The robot’s right-turn angular velocity is also set as positive. Let θ denote the wrist angle. The robot’s angular velocity ω is mapped as

(2) $$\omega =\left\{\begin{array}{cc} \frac{\theta +\theta_{\mathrm{LD}}}{\theta_{\mathrm{LM}}-\theta_{\mathrm{LD}}}\cdot \omega_{\max} & \mathrm{if}\;-\theta_{\mathrm{LM}}<\theta <-\theta_{\mathrm{LD}}\\ 0 & \mathrm{if}\;-\theta_{\mathrm{LD}}<\theta <\theta_{\mathrm{RD}}\\ \frac{\theta -\theta_{\mathrm{RD}}}{\theta_{\mathrm{RM}}-\theta_{\mathrm{RD}}}\cdot \omega_{\max} & \mathrm{if}\;\theta_{\mathrm{RD}}<\theta <\theta_{\mathrm{RM}} \end{array}\right.$$

where $\theta_{\mathrm{LM}}$ and $\theta_{\mathrm{RM}}$ are the maximum wrist extension and flexion angles, while $\theta_{\mathrm{LD}}$ and $\theta_{\mathrm{RD}}$ define the deadzone, suppressing noise when the hand is in a neutral position. $\omega_{\max}$ represents the robot’s maximum angular velocity.

The corresponding linear velocity is given by

(3) $$v=v_{\max}\cdot \left(1-\frac{\left|\omega \right|}{\omega_{\max}}\right)$$

where $v_{\max}$ is the robot’s maximum linear velocity. Maximum linear velocity occurs during straight-line motion (ω=0), decreasing to zero during maximum-rate turns ($\omega =\pm \omega_{\max}$).

### 2.3. Data Collection

This study was conducted in accordance with the Declaration of Helsinki and approved by the Institutional Review Board of Northwestern Polytechnical University (protocol code No. 202302026 in 2023). Informed consent was obtained from all participants. A total of 15 healthy adults (12 males, 3 females) were recruited, with a mean age of 25.3 ± 3.1 years (range 21–32). Inclusion criteria were normal or corrected-to-normal vision, absence of neuromuscular disorders, and no history of allergic reactions to EMG electrodes. All participants were right-handed; the experimental task was performed using the dominant right hand for wrist movements, with the EMG armband worn on the left forearm. This non-dominant hand control was designed to simulate the scenario of amputees using residual limb muscles to control prosthetic devices.

sEMG data were collected using the ELONXI system (Hangzhou Jiaopu Technology Co., Ltd., Hangzhou, China) at a 1000 Hz sampling rate. The device supports up to 16 channels, transmitting data to the control platform via Bluetooth. This study employed an 8-channel armband configuration, with sensors uniformly distributed around the forearm to capture wrist flexion/extension activation patterns. The 8-channel setup balances muscle coverage with computational efficiency and reduced cross-talk.

**Figure 2:**
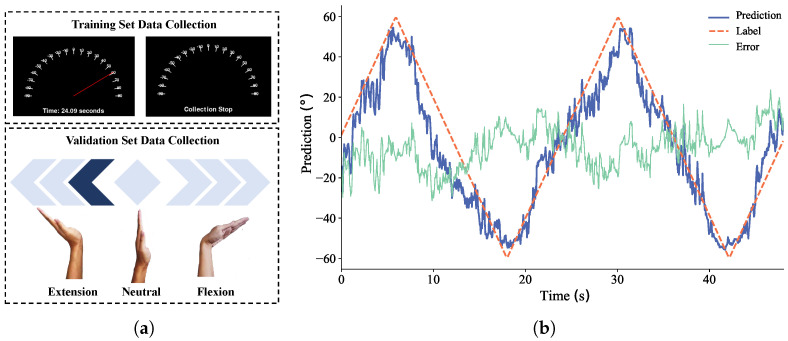
(**a**) Animation of training set (**top**) and validation set (**bottom**) for indicating wrist flexion-extension angles. (**b**) RF regression prediction results. The orange dashed line represents the labels, the blue solid line represents the predictions, and the green solid line represents the error.

As no dedicated wrist angle sensors [29] were used, we designed two animated cues to guide participants in performing the required movements (Figure 2a). During the collection of sEMG data for training the motion estimation model, participants were instructed to maintain a palm-facing gesture and follow the animation in Figure 2a, where the pointer oscillated back and forth, guiding wrist motion. The angular velocity of the pointer in the animation remained constant at 10° per second, providing guidance on the wrist’s range of motion and simultaneously labeling wrist flexion and extension angles.

For the data collection used to validate the model, participants were required to adjust their wrist angles according to the prompts in Figure 2a. Given the inherent imprecision in human movement, the wrist’s extension or flexion was divided into three amplitude levels, leading to seven wrist joint angle intervals: $\left[-\phi_{\mathrm{LM}},-\frac{5}{7}\phi_{\mathrm{LM}}\right)$, $\left[-\frac{5}{7}\phi_{\mathrm{LM}},-\frac{3}{7}\phi_{\mathrm{LM}}\right)$, $\left[-\frac{3}{7}\phi_{\mathrm{LM}},-\frac{1}{7}\phi_{\mathrm{LM}}\right)$, $\left[-\frac{1}{7}\phi_{\mathrm{LM}},\frac{1}{7}\phi_{\mathrm{RM}}\right)$, $\left[\frac{1}{7}\phi_{\mathrm{RM}},\frac{3}{7}\phi_{\mathrm{RM}}\right)$, $\left[\frac{3}{7}\phi_{\mathrm{RM}},\frac{5}{7}\phi_{\mathrm{RM}}\right)$, and $\left[\frac{5}{7}\phi_{\mathrm{RM}},\phi_{\mathrm{RM}}\right]$, where $\phi_{\mathrm{LD}}=\frac{1}{7}\phi_{\mathrm{LM}}$ and $\phi_{\mathrm{RD}}=\frac{1}{7}\phi_{\mathrm{RM}}$. Deep blue arrows indicated different angular intervals, and their direction represented wrist movement. Participants were instructed to quickly position their wrist within the specified angular interval as soon as the action in the video changed. The task lasted for one minute, with each target angle maintained for 5 s, and 12 angle transitions in total. The training animation naturally covers each participant’s comfortable range, which sets the individualized $\theta_{\mathrm{LM}}$ and $\theta_{\mathrm{RM}}$ in Equation (2).

All experiments in this work were conducted on a computer running Ubuntu 18.04, equipped with an Intel i7-12700 CPU and 32 GB of RAM.

### 2.4. Feature Extraction

To mitigate industrial frequency interference and preserve relevant signal components [30,31], we applied a bandpass Butterworth filter (15–480 Hz) and a 50 Hz notch filter to denoise the sEMG signals. Feature extraction was performed using a sliding window of 250 ms with a 50 ms step size.

We analyzed ten common time-domain features—Root Mean Square (RMS), Mean Absolute Value (MAV), Waveform Length (WL), Zero Crossing (ZC), Slope Sign Changes (SSC), Variance (VAR), Simple Square Integral (SSI), Maximum (MAX), Log-Detector (LogD), and Willison Amplitude (WAMP)—alongside six frequency-domain features: Slow Feature Analysis (SFA), Median Frequency (MF), DC Component (F-DC), Mean Frequency Spectrum (F-M), Standard Deviation of Frequency Spectrum (F-STD), and Entropy of Frequency Spectrum (F-ENT).

Spearman’s rank correlation analysis was used to assess feature redundancy, identifying a subset with relatively low correlation. Balancing recognition accuracy and computational efficiency, we selected a hybrid feature set comprising WL, WAMP, and F-ENT.

### 2.5. Decoding Model

RF regression method are widely recognized for their robust performance in prediction accuracy and processing speed compared to other machine learning methods [5,14,15,16]. Leveraging this, we trained an RF regression model using the selected hybrid feature set (WL, WAMP, F-ENT) to estimate wrist joint motion during myoelectric control.

The dataset was split into 80% for training and 20% for testing. Figure 2b shows the prediction results for a test dataset with $\phi_{\mathrm{RM}}=\phi_{\mathrm{LM}}=60^{\circ }$. The RF model’s predictions of wrist flexion–extension angles closely align with the reference values, with errors generally within 20°, demonstrating its potential for wrist angle prediction. However, high-frequency fluctuations in the predicted joint angles introduce noise, causing the prediction curve to oscillate around the target. Therefore, post-processing of the regression is necessary to enhance stability.

## 3. Proposed Method

In continuous wrist joint motion estimation, instantaneous regression outputs often exhibit temporal fluctuations due to sEMG signal noise, muscle fatigue, and feature distribution shifts. Although the RF model provides accurate nonlinear mapping from feature vector $x_{t}$ to joint angle ${\hat{\theta }}_{t}$, its predictions may lack temporal consistency. Traditional post-processing approaches such as moving average [22] or exponential smoothing [26] apply fixed filtering strength and do not account for prediction confidence.

To address this limitation, we reformulate post-processing as an uncertainty-aware probabilistic fusion problem. Instead of embedding Bayesian inference into the regression backbone, we decouple prediction and uncertainty estimation. The RF model provides instantaneous prediction, while a LGPR model constructed on recent smoothed outputs provides both a local temporal prior and predictive uncertainty. These two sources are fused under a probabilistic framework to produce the final smoothed output ${\tilde{\theta }}_{t}$.

### 3.1. Sliding Window Dataset Construction

At time step *t*, the feature vector extracted from sEMG signals is denoted as $x_{t}$, and the RF prediction is

(4) $${\hat{\theta }}_{t}=\mathrm{RF}\left(x_{t}\right).$$

To capture short-term temporal dynamics, we maintain a sliding window dataset

(5) $$D_{t}={\left\{\left(x_{i},{\tilde{\theta }}_{i}\right)\right\}}_{i=t-N+1}^{t-1},$$

where ${\tilde{\theta }}_{i}$ denotes previously smoothed predictions. The window size *N* is fixed to ensure computational efficiency. After computing the current fused output ${\tilde{\theta }}_{t}$, the dataset is updated as

(6) $$D_{t}=D_{t}\cup \left\{\left(x_{t},{\tilde{\theta }}_{t}\right)\right\},$$

and the oldest sample is removed if necessary to maintain constant size.

### 3.2. LGPR Modeling

Using the sliding dataset $D_{t}$, we construct a Local Gaussian Process Regression model. Let the kernel matrix over the window be

(7) $$K_{ij}=k\left(x_{i},x_{j}\right),$$

where the Matérn kernel [32] is adopted in our implementation. For a new input $x_{t}$, define the kernel vector

(8) $$k\left(x_{t}\right)={\left[k\left(x_{t},x_{t-1}\right),\ldots ,k\left(x_{t},x_{t-N+1}\right)\right]}^{⊤}.$$

The LGPR predictive mean is

(9) $${\hat{\theta }}_{t}^{GP}=k{\left(x_{t}\right)}^{⊤}{\left(K+\sigma_{n}^{2}I\right)}^{-1}y_{t},$$

where $y_{t}={\left[{\tilde{\theta }}_{t-1},{\tilde{\theta }}_{t-2},\ldots ,{\tilde{\theta }}_{t-N+1}\right]}^{⊤}$. The predictive variance is

(10) $$\Sigma_{t}=k\left(x_{t},x_{t}\right)-k{\left(x_{t}\right)}^{⊤}{\left(K+\sigma_{n}^{2}I\right)}^{-1}k\left(x_{t}\right).$$

Here, ${\hat{\theta }}_{t}^{GP}$ captures the locally consistent motion trend inferred from recent outputs, while $\Sigma_{t}$ quantifies predictive uncertainty. Notably, $\Sigma_{t}$ increases when the current feature vector lies in sparsely supported regions or during abrupt motion transitions.

### 3.3. Theoretical Justification for Uncertainty Proxy

Although $\Sigma_{t}$ is an output of the LGPR model, its physical interpretation is out-of-distribution detection in the feature space. The core assumption is that RF and LGPR share the same input feature space (WL, WAMP, and F-ENT).

When the input feature $x_{t}$ falls into a sparse region of the training distribution (e.g., during rapid motion transitions where muscle activation patterns change abruptly), the Matérn kernel $k(x_{t})$ produces small kernel values, leading to increased predictive variance $\Sigma_{t}$. This anomaly in the feature space propagates directly to the RF prediction, since RF is also trained on the historical feature-to-angle mapping, distribution shift inevitably degrades prediction reliability.

Therefore, $\Sigma_{t}$ captures anomaly in the feature space, which is precisely the dominant source of RF prediction error. The connection between the two is established through distribution shift in the feature space, rather than direct assessment of RF by LGPR.

### 3.4. Uncertainty-Aware Probabilistic Fusion

We interpret the instantaneous RF prediction as a noisy observation:

(11) $$\theta_{t}^{obs}∼N\left({\hat{\theta }}_{t},\Sigma_{t}\right),$$

where $\Sigma_{t}$ serves as a data-driven confidence measure. Meanwhile, the LGPR prediction is treated as a prior belief:

(12) $$\theta_{t}^{prior}∼N\left({\hat{\theta }}_{t}^{GP},\sigma_{p}^{2}\right),$$

where $\sigma_{p}^{2}$ controls temporal regularization strength. Under Gaussian assumptions and conditional independence, the posterior mean is obtained analytically as

(13) $${\tilde{\theta }}_{t}=\frac{{\hat{\theta }}_{t}/\Sigma_{t}+{\hat{\theta }}_{t}^{GP}/\sigma_{p}^{2}}{1/\Sigma_{t}+1/\sigma_{p}^{2}}.$$

This can be rewritten in gain form:

(14) $$G_{t}=\frac{1/\Sigma_{t}}{1/\Sigma_{t}+1/\sigma_{p}^{2}}=\frac{\sigma_{p}^{2}}{\Sigma_{t}+\sigma_{p}^{2}},$$

(15) $${\tilde{\theta }}_{t}=G_{t}{\hat{\theta }}_{t}+\left(1-G_{t}\right){\hat{\theta }}_{t}^{GP}.$$

Therefore, the final estimate is a convex combination of instantaneous prediction and local temporal prior, with adaptive gain determined by predictive uncertainty. The complete implementation procedure of the proposed framework is summarized in Algorithm 1.

The adaptive gain satisfies $0<G_{t}<1$ for all $\Sigma_{t}>0$, ensuring that ${\tilde{\theta }}_{t}$ remains bounded between ${\hat{\theta }}_{t}$ and ${\hat{\theta }}_{t}^{IGP}$. Taking derivative with respect to $\Sigma_{t}$,

(16) $$\frac{\partial G_{t}}{\partial \Sigma_{t}}=-\frac{\sigma_{p}^{2}}{{\left(\Sigma_{t}+\sigma_{p}^{2}\right)}^{2}}<0,$$

which proves strict monotonic decrease of $G_{t}$ with uncertainty. Thus, smoothing strength increases consistently as confidence decreases. Furthermore,

(17) $$\lim_{\Sigma_{t}\to 0}G_{t}=1,\;\lim_{\Sigma_{t}\to \infty }G_{t}=0,$$

indicating that the framework smoothly interpolates between instantaneous prediction and temporal prior.

Since the fused output is bounded and the sliding window size is finite, the overall system is bounded-input bounded-output stable provided the RF outputs remain bounded. Finally, the update structure is mathematically equivalent to a one-step Bayesian update with measurement variance $\Sigma_{t}$ and prior variance $\sigma_{p}^{2}$, resembling a Kalman-like gain without requiring an explicit state transition model.

In the overall control pipeline (see Figure 1), the proposed probabilistic fusion framework operates as a post-processing module after the regression-based wrist angle estimation. The refined wrist angle estimate ${\tilde{\theta }}_{t}$ is subsequently converted into robot motion commands through the parameter mapping defined in Equations (2) and (3), generating the linear and angular velocities used for robot control.
**Algorithm 1** Uncertainty-Aware Probabilistic Fusion Post-Processing**Input:** Trained regression model RF(·); Sliding window size *N*; Prior variance $\sigma_{p}^{2}$**Output:** Smoothed wrist angle estimate $\left\{{\tilde{\theta }}_{t}\right\}$  1:  Initialize $D_{t}\leftarrow \emptyset$  2:  **for** each *t* **do**  3:        Extract sEMG feature vector $x_{t}$  4:        Compute instantaneous prediction: ${\hat{\theta }}_{t}=\mathrm{RF}\left(x_{t}\right)$  5:        **if** $|D_{t}|\;<\;N$ **then**  6:              ${\tilde{\theta }}_{t}\leftarrow {\hat{\theta }}_{t}$  7:        **else**  8:              Construct LGPR using dataset $D_{t}$  9:              Compute $\left({\hat{\theta }}_{t}^{GP},\Sigma_{t}\right)$ using Equations (7)–(10)10:              Compute adaptive gain: $G_{t}=\frac{\sigma_{p}^{2}}{\Sigma_{t}+\sigma_{p}^{2}}$11:              Perform probabilistic fusion: ${\tilde{\theta }}_{t}=G_{t}{\hat{\theta }}_{t}+\left(1-G_{t}\right){\hat{\theta }}_{t}^{GP}$12:        **end if**13:        Update $D_{t}$ while keeping size *N*14:  **end for**

## 4. Results and Analysis

To validate the proposed method, we first conducted regression predictions on continuous gesture data in an open-loop setting, without robot involvement. We then designed two closed-loop myoelectric control tasks to evaluate control performance. All experiments were performed with fifteen colleagues from the author’s laboratory.

Unless otherwise specified, the parameters used in the experiments are set as follows. The sEMG feature extraction uses a window length of 250 ms with a step size of 50 ms. For LGPR uncertainty estimation, the window size is set to N=30. The observation noise variance in the probabilistic fusion model is set to $\sigma_{p}^{2}=0.01$. For the RF regression model, the number of trees is set to 100, and the maximum tree depth is set to 15. For the mobile robot control, the maximum linear velocity is limited to $v_{\max}=0.3\;m/s$ and the maximum angular velocity is limited to $\omega_{\max}=1.0\;\mathrm{rad}/s$. These parameters were empirically determined to balance prediction stability and computational efficiency in real-time control. The chosen values ensure closed-loop control at 10 Hz without frame drops (Section 4.3), which is the primary deployability criterion for this application.

### 4.1. Review of Existing Post-Processing Methods

Various post-processing methods have been reported in the literature with some success. Below, we summarize some of the representative methods:

*Moving Average (MA)* [24]: A straightforward time-series smoothing technique that averages each data point with its neighbors. The smoothed prediction is given by ${\tilde{\theta }}_{t}=\frac{1}{m}\sum_{k=0}^{m-1}{\hat{\theta }}_{t-k}$, where *m* is the length of the sliding window. A larger *m* results in smoother predictions, but too large an *m* introduces time lag.

*Exponential Moving Average (EMA)* [25]: A weighted moving average method that gives more weight to recent data points. The formula is ${\tilde{\theta }}_{t}=\lambda {\tilde{\theta }}_{t-1}+\left(1-\lambda \right){\hat{\theta }}_{t}$, where λ balances smoothing capability and time delay. A larger λ results in greater smoothing but introduces more lag. In our implementation, λ was set to 0.7.

*Median Filtering (MF)* [22]: Replaces each data point with the median of neighboring values within a sliding window, effectively reducing noise and outliers.

*Savitzky–Golay Filter (SG)* [23]: Fits a polynomial to a local window of data points, preserving trends while smoothing the signal.

*Wavelet Denoising (WD)* [26]: Decomposes signals into different frequency components via wavelet transform, applying thresholding to suppress noise while retaining key signal features.

### 4.2. Open-Loop Continuous Wrist Motion Estimation

We compared the proposed method (LGPR) with the five post-processing schemes introduced in Section 4.1, as well as a baseline RF regression model without post-processing. RF was selected as the base regression model due to its strong performance and computational efficiency in sEMG-based motion decoding reported in previous studies [5,14,15,16]. Since the primary objective of this work is to investigate probabilistic post-processing strategies for stabilizing regression outputs, the underlying regression model was kept consistent across all methods to ensure a fair comparison.

Since wrist joint angle predictions in the validation set are assessed based on whether they fall within a specified range rather than their absolute accuracy against ground truth labels, traditional regression metrics such as RMSE and $R^{2}$ are not suitable. Instead, we use the hit rate as a measure of the regression prediction accuracy. The hit rate is defined as the probability that the predicted value falls within the specified angle range and is given by

(18) $$R_{\mathrm{hit}}=\frac{1}{n}\sum_{i=1}^{n}I\left({\tilde{\theta }}_{i}\right)$$

where *n* is the number of samples to be estimated, and I(·) is the indicator function, which equals 1 if the prediction is within the range, and 0 otherwise.

In addition, we introduce another metric to quantify the smoothness of joint motion, denoted as $S_{\mathrm{wrist}}$:

(19) $$S_{\mathrm{wrist}}=\frac{1}{n-1}\sum_{i=1}^{n-1}\left|{\tilde{\theta }}_{i+1}-{\tilde{\theta }}_{i}\right|$$

where a smaller value of $S_{\mathrm{wrist}}$ indicates greater smoothness.

**Figure 3:**
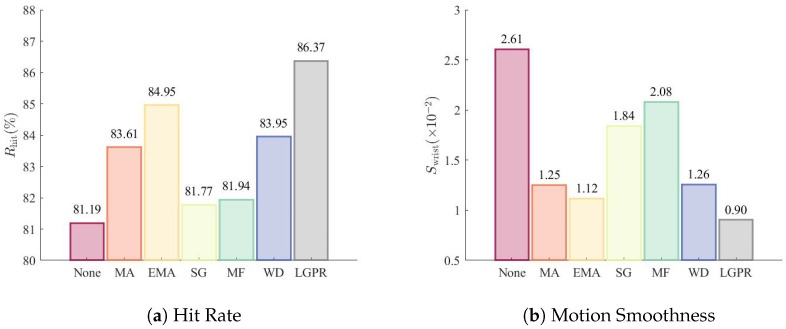
Comparison of seven post-processing methods for continuous wrist motion estimation.

Figure 3 compares the hit rate and joint motion smoothness obtained with different post-processing methods. The proposed method achieves the highest hit rate, followed by EMA. Among the five post-processing schemes introduced in Section 4.1, EMA remains the most effective and robust method overall despite its simple computational structure.

**Figure 4:**
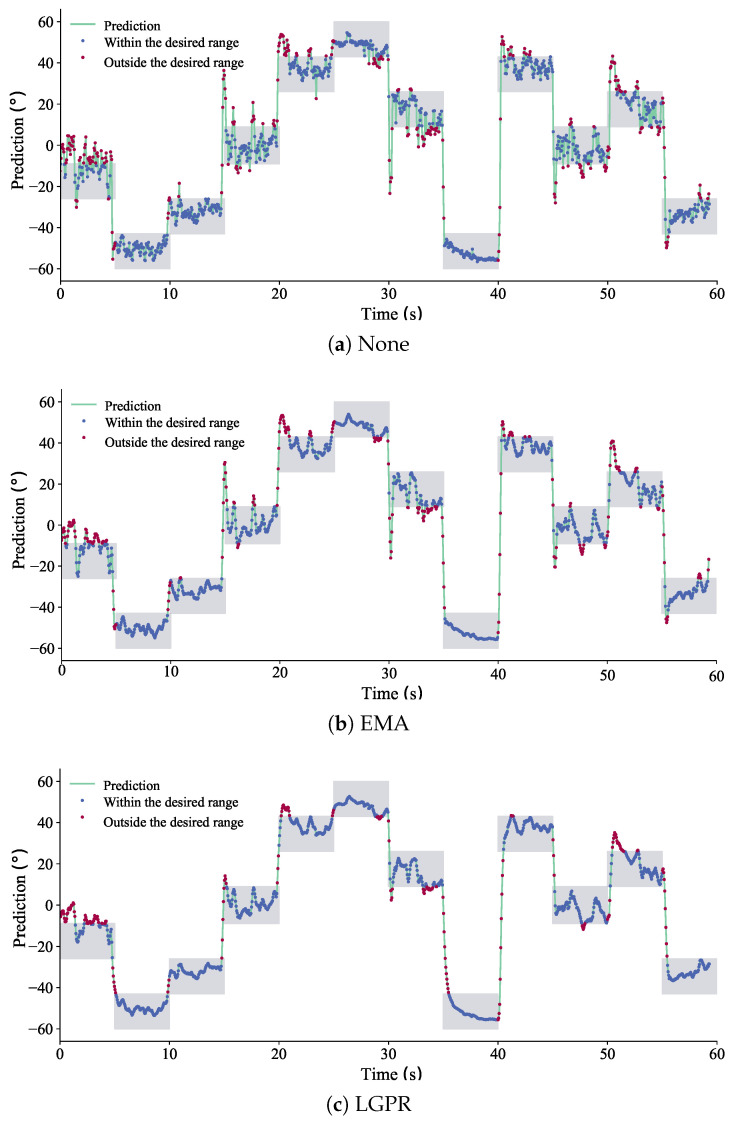
Wrist joint angle regression results using EMA, LGPR (Ours), and no post-processing (None). The gray area represents the desired wrist joint angle range, blue dots indicate predictions within the desired range, and red dots indicate predictions outside the desired range.

Figure 4 illustrates representative wrist joint angle regression results using EMA post-processing, LGPR post-processing, and no post-processing. Although the RF regression model without post-processing generally produces predictions within the expected angle range, noticeable fluctuations are present in the predicted trajectories. In particular, large instantaneous deviations often occur during wrist angle transitions. These deviations are mainly caused by rapid changes in muscle stiffness when the wrist motion switches between different postures, and the phenomenon becomes more pronounced in tasks involving frequent motion transitions.

EMA reduces both global and local fluctuations in the prediction sequence through fixed-weight smoothing that inevitably introduces phase lag. Its ability to handle erroneous predictions during motion transitions remains limited—the constant smoothing strength cannot distinguish between high-confidence steady-state predictions and uncertain transition dynamics. In particular, “spike” signals may still appear at transition points, and the uniform filtering delay can accumulate across the control pipeline, degrading the stability of myoelectric control.

In contrast, the proposed LGPR-based method achieves stronger smoothing performance, effectively suppressing most transition-induced spikes without introducing systematic phase lag. The state-dependent gain mechanism (Equation (12)) ensures that: (i) during steady-state periods with low predictive uncertainty ($\Sigma_{t}\to 0$), the gain $G_{t}\to 1$ and the output follows the instantaneous prediction with minimal delay; (ii) during high-uncertainty transitions, the reduced gain provides targeted smoothing only where needed. This adaptive latency—fast when confident, cautious when uncertain—preserves the real-time characteristics required for online motion estimation, as visually confirmed by the tight tracking of reference trajectories in Figure 4c.

### 4.3. Closed-Loop Myoelectric Control

**Figure 5:**
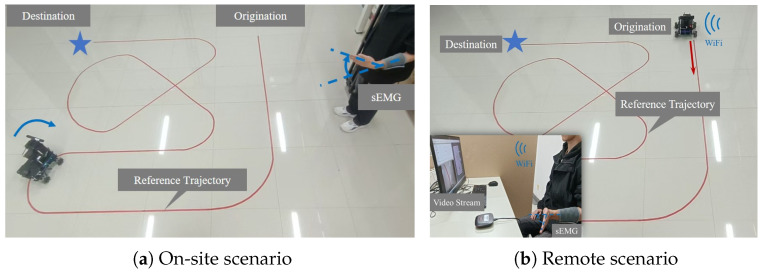
Closed-loop myoelectric control setup. The red solid line represents the reference trajectory to be followed. (**a**) The operator perceives the robot’s state on-site to control its motion. (**b**) The operator, using images from an onboard camera, observes the robot’s position relative to the reference trajectory and adjusts the robot’s motion accordingly.

A task scenario was designed (see Figure 5) in which the operator controlled a ground robot to follow a predefined reference trajectory from the start point to the destination using sEMG signals generated by wrist movements. The experiment was conducted under two conditions: on-site control and remote control.

Fifteen participants tested three myoelectric control methods: with EMA post-processing, with LGPR post-processing, and without post-processing, each tested once. To minimize learning effects, participants completed a 15-min familiarization session before formal data collection, including 2–3 practice trials for each condition; practice data were not analyzed. The presentation order of the three control methods was counterbalanced across participants using a Latin square design to eliminate ordering and carryover effects.

During the experiments, task completion time, robot position, robot state, and online recognition results before and after post-processing were recorded. Consequently, 15 sets of experimental data were obtained for each scenario.

Three performance metrics were evaluated:

- *Task Completion Time $T_{\mathrm{task}}$*: the time required for the robot to move from the start point to the destination.
- *Robot Trajectory Smoothness $S_{\mathrm{traj}}$*: computed as the average curvature difference between adjacent time steps during the motion. A smaller value indicates a smoother trajectory.
- *Trajectory Tracking Error $E_{\mathrm{traj}}$*: calculated as the average shortest distance from each point on the robot trajectory to the reference trajectory. A smaller value indicates better tracking performance.

Statistical comparisons were performed using one-way repeated-measures ANOVA, with Greenhouse–Geisser correction for violations of sphericity. Post hoc pairwise comparisons were conducted using paired *t*-tests with Bonferroni correction, and $\eta_{p}^{2}$ was reported as the effect size. All statistical analyses were performed in SPSS (v26.0), with a significance level of p=0.05.

**Figure 6:**
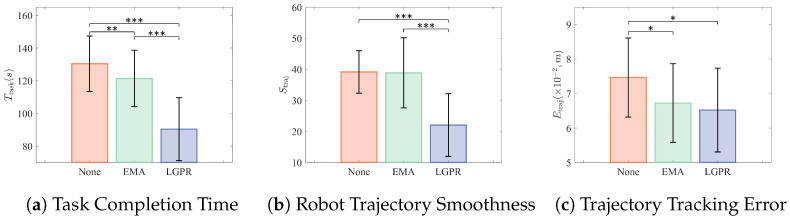
Performance metrics of myoelectric control in the on-site condition. Significance markers: * *p* < 0.05, ** *p* < 0.01, *** *p* < 0.001.

**Figure 7:**
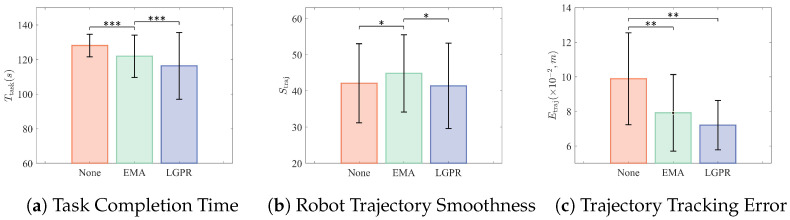
Performance metrics of myoelectric control in the remote condition. Significance markers: * *p* < 0.05, ** *p* < 0.01, *** *p* < 0.001.

Results of the closed-loop myoelectric control tasks in the on-site and remote conditions are illustrated in Figure 6 (on-site) and Figure 7 (remote), respectively. Statistical analysis was performed using repeated-measures comparisons with Bonferroni correction.

In the on-site condition, a significant main effect of method was identified for task completion time (F(2,28)=68.62, p<0.001, $\eta_{p}^{2}=0.833$). Pairwise comparisons showed significant differences across all groups: no post-processing vs. EMA (p=0.003), no post-processing vs. LGPR (p<0.001), and EMA vs. LGPR (p<0.001).

For trajectory smoothness, a significant main effect was also observed (F(2,28)=21.45, p<0.001, $\eta_{p}^{2}=0.607$), and the LGPR method achieved significantly smoother trajectories than both the no post-processing condition (p<0.001) and EMA (p<0.001).

In terms of trajectory tracking error, a significant main effect of method was detected (F(2,28)=3.86, p=0.034, $\eta_{p}^{2}=0.216$). Both LGPR and EMA exhibited significantly smaller errors than the no post-processing condition (LGPR: p=0.026; EMA: p=0.041), whereas no significant difference was found between LGPR and EMA.

In the remote condition, a significant main effect of method was found for task completion time (F(2,28)=19.87, p<0.001, $\eta_{p}^{2}=0.587$). The LGPR method was significantly faster than both the no post-processing condition (p<0.001) and EMA (p<0.001), while no significant difference was observed between the no post-processing condition and EMA.

For trajectory smoothness, a significant main effect was observed (F(2,28)=4.76, p=0.017, $\eta_{p}^{2}=0.254$), and LGPR provided significantly smoother trajectories than both the no post-processing condition (p=0.013) and EMA (p=0.029).

For trajectory tracking error, a significant main effect of method was identified (F(2,28)=8.92, p=0.001, $\eta_{p}^{2}=0.390$). Both LGPR and EMA showed significantly lower errors than the no post-processing condition (LGPR: p=0.002; EMA: p=0.006), and no significant difference was observed between LGPR and EMA.

Overall, the proposed LGPR method achieved superior performance in both on-site and remote conditions, with significantly shorter task completion time, smoother trajectories, and better tracking accuracy compared with conventional methods.

**Figure 8:**
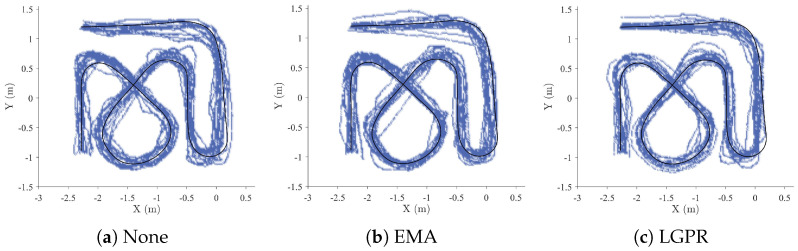
Sample trajectories from three methods in on-site scenarios. The black solid line represents the reference trajectory, and the blue portion indicates regions of the robot’s state space with higher visit frequency.

**Figure 9:**
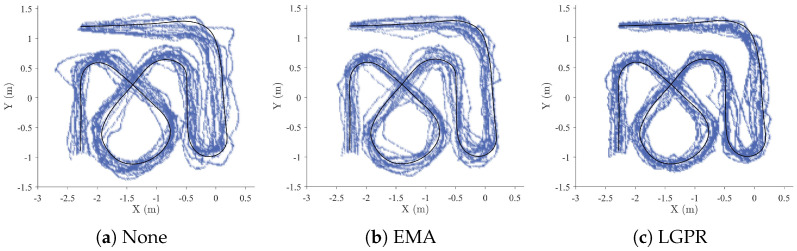
Sample trajectories from three methods in remote scenarios. The black solid line represents the reference trajectory, and the blue portion indicates regions of the robot’s state space with higher visit frequency.

Figure 8 and Figure 9 illustrate the distribution of robot trajectories, high-frequency visited regions, and deviations from the reference trajectory. The trajectories generated using the proposed method are smoother and more concentrated around the reference path compared to EMA and the method without post-processing. In the remote scenario, trajectories produced by all methods exhibit some deviation from the reference path; however, post-processed control results in noticeably smoother trajectories. In the on-site scenario, deviations from the reference trajectory are generally smaller, as the operator can directly observe the robot’s motion state and adjust control actions accordingly.

Overall, regardless of whether the operator was on-site or remote, myoelectric control with post-processing produced trajectories with smaller fluctuations and reduced deviation from the reference trajectory, demonstrating improved stability and consistency.

### 4.4. Discussion

The proposed uncertainty-aware probabilistic fusion post-processing framework effectively improves the stability of continuous wrist motion estimation from sEMG signals. Different from conventional regression models [1,14,19] that only provide point predictions, our method estimates prediction uncertainty by measuring feature distribution consistency in a local sliding window. This uncertainty reflects out-of-distribution inputs in the shared feature space, rather than model internal confidence, and thus provides a calibrated basis for adaptively adjusting smoothing strength. This mechanism avoids the fixed-weight limitation of traditional smoothing filters and is more suitable for non-stationary and signal-dependent noise in sEMG control.

Experimental results show that the proposed method significantly improves trajectory smoothness, hit rate, task completion speed, and tracking accuracy compared with conventional post-processing methods. The uncertainty-aware fusion strategy suppresses transient fluctuations and transition spikes while maintaining response speed, which enhances the robustness and practicality of myoelectric control. Meanwhile, the sliding-window online update design keeps computational cost low and enables real-time performance for embedded robotic control systems [24].

Several limitations exist in this study. First, the wrist angle ground truth is provided by animated cues instead of physical sensors, which supports functional task evaluation but restricts precise kinematic analysis. Second, although the adaptive smoothing strategy effectively balances stability and responsiveness, quantitative latency and phase-lag analysis will be included in future work to clarify the smoothness–delay–accuracy trade-off. Third, the proposed framework is validated only on a random forest backbone; although it is structurally compatible with various regression models, validations on more regressors will further demonstrate its generalization ability.

It should be emphasized that the proposed method is not equivalent to standard Kalman-like filtering, since it does not rely on a predefined state transition model or fixed noise parameters, making it more practical for real-world sEMG scenarios. In addition, the method is inherently robust to signal-dependent noise that changes with muscle activation and fatigue, because the uncertainty module automatically captures feature instability and adjusts smoothing strength accordingly [28].

The current single-degree-of-freedom platform serves as a valid test bed for the proposed method. In the future, the framework will be extended to multi-degree-of-freedom motion estimation [3,18]. Furthermore, intuitive control using virtual stiffness estimated from muscle co-contraction will be explored to achieve more natural and decoupled human-robot interaction [5,12,22].

## 5. Conclusions

This paper presents an uncertainty-aware probabilistic fusion post-processing framework for continuous wrist joint motion estimation from sEMG signals. By decoupling regression prediction from uncertainty modeling and employing LGPR to estimate predictive variance, our framework enables adaptive confidence-aware smoothing through a closed-form gain formulation. Experimental results from both open-loop motion estimation and closed-loop myoelectric control tasks demonstrate that the proposed approach improves trajectory smoothness, tracking accuracy, and task efficiency compared with conventional post-processing methods. Future work will focus on extending the framework to multi-DoF motion estimation and exploring its integration with deep neural network predictors. Further investigation will also consider improving computational efficiency and evaluating the method in more complex real-world environments.

## Data Availability

The raw data supporting the conclusions of this article will be made available by the authors on request.
